# Establishing a Digital Health Care Ecosystem in a Health Sciences University in South Africa: Protocol for a Mixed Methods Study

**DOI:** 10.2196/57821

**Published:** 2025-03-25

**Authors:** Samantha Govender, Maria Elizabeth Cochrane, Mabina Mogale, Reno Gordon, Tjodwapi Tshephe

**Affiliations:** 1 Department of Speech and Language Pathology and Audiology School of Health Care Sciences Sefako Makgatho Health Sciences University Pretoria South Africa; 2 Faculty of Health Sciences Health Professions Education University of Pretoria Pretoria South Africa; 3 Department of Public Health School of Health Care Sciences Sefako Makgatho Health Sciences University Nay Pyi Taw Myanmar; 4 Department of Human Nutrition School of Health Care Sciences Sefako Makgatho Health Sciences University Pretoria South Africa; 5 School of Health Care Sciences Sefako Makgatho Health Sciences University Pretoria South Africa

**Keywords:** health sciences, digital ecosystem, curriculum, community engagement, tertiary education institutions

## Abstract

**Background:**

Comprehensive and formalized digital health care ecosystems in health sciences tertiary education in South Africa do not currently exist, but they have the potential to influence teaching and learning, research, and community engagement.

**Objective:**

A total of 3 key objectives underpin the study, that is, determining the health care curriculum needs and required content for the development of a formalized digital health ecosystem, determining the level of readiness of staff and students to participate in a digital health care ecosystem, and determining whether community engagement and strategic partnerships can contribute to the sustainability of a digital health care ecosystem.

**Methods:**

A multipronged approach will be used to address the objectives, with a mixed methods design being undertaken. The qualitative phases will be phenomenological in nature, and triangulation of information along with thematic analysis will be conducted on the collected data. Quantitative data will be collected prospectively and cross-sectionally and analyzed using descriptive analysis. Sampling will include subject experts for the Delphi technique, staff and students at the University, clinical training and education partners, and community leaders. This study has received ethical approval from the Sefako Makgatho Health Sciences University Research and Ethics Committee (SMUREC/H/260/2023:PG).

**Results:**

Data collection for the first phase will begin in January 2024 and conclude in December 2024. Phase 2 and 3 of the study will be conducted concurrently, with data collection starting in January 2025 and concluding in December 2026.

**Conclusions:**

The establishment of a digital health care ecosystem has the potential to benefit staff, students, and communities through stakeholder collaboration, educational opportunities, research projects, and improved service delivery.

**International Registered Report Identifier (IRRID):**

DERR1-10.2196/57821

## Introduction

Digital health care ecosystems are described as patient-centric models of health care delivery with the aim of encouraging collaborative, cross-organizational health care processes through technology-driven platforms [1]. The use of telecommunications and digital technologies to provide health care, monitor public health, and develop new and improved methods of delivering awareness programs is referred to as digital health care [2]. According to the World Health Organization (WHO) [2], “digital health” is a global term that encompasses concepts such as electronic health, telehealth, wearable devices, mobile health, and new developments in the field of artificial intelligence to support public health systems. The definition also includes the use of electronic patient and medical record keeping.

The benefits of digital health care are extensive, including serving populations in rural and remote areas with interventional and preventative care [3], the collation and pooling of medical data, and the organized and confidential management of patient records [2]. However, despite these benefits, there is evidence to suggest that health care professionals (HCPs) are inadequately trained to effectively use technology within the health care space [4-6]. This issue is especially true in the African context [7]. Despite Africa accounting for 23% of the global burden of disease, the continent has not optimized digital health care technologies to address challenges in health care service delivery [7]. South Africa has been passive in its efforts to ensure that HCPs are adequately trained. The COVID-19 pandemic highlighted the lack of preparedness in digital health care, which has been attributed to insufficient exposure to digital health care training during the tertiary education training of HCPs [8,9]. This is important because the lack of digital health care competencies and skills could pose significant challenges to the health care industry. Potential challenges that may arise from a lack of digital health care skills include reduced efficiency [10], ineffective use of digital health care tools [11], reduced patient satisfaction due to low HCP confidence [12], increased risk of errors [13], and lack of globally relevant skills [7].

**Figure 1:**
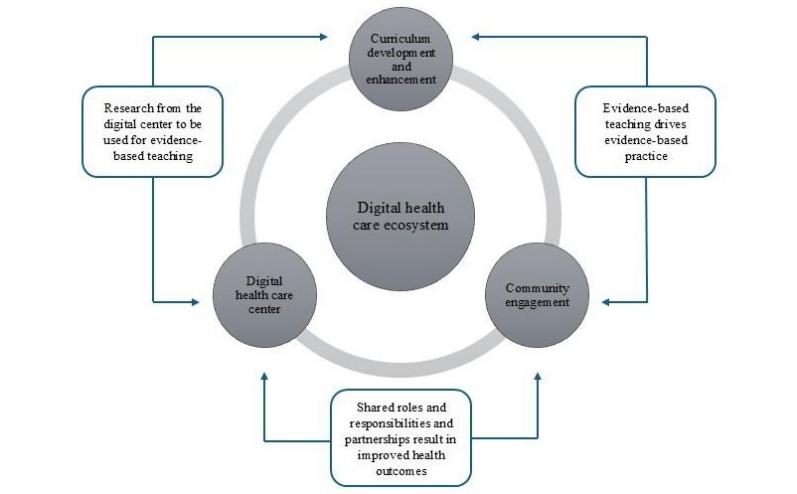
Digital health care ecosystem.

Challenges faced by the health care system and universities during the pandemic were threefold [8,14,15]. First, staff and students were not well trained or competent in providing digital health care to patients. Second, some universities were not well invested in digital health care infrastructure. Third, the acceptance of communities to receive care via digital technologies was uncertain, considering the lack of consultation and stakeholder engagement [8,14,15]. It becomes apparent that countries, through their health care systems and tertiary training institutions, need to do the following three things: First, create, construct, and integrate curricula that align with the needs of a new era of HCPs. Second, provide the necessary infrastructure to allow student learning and clinical training opportunities within the digital health care space. Finally, to actively engage with communities to ensure their integration, involvement, and acceptance of digital health care services. The combination of these 3 elements creates what is referred to as a digital health care ecosystem [16]. Therefore, the objectives of the study were threefold: first, determining the health care curriculum needs and required content for the development of a formalized digital health ecosystem; second, determining the level of readiness of staff and students to participate in a digital health care ecosystem; and third, determining whether community engagement and strategic partnerships can contribute to the sustainability of a digital health care ecosystem. Figure 1 illustrates the envisaged digital health care ecosystem (Figure 1).

## Methods

### Study Design

A multiphase study, consisting of a mixed methods approach (ie, qualitative and quantitative designs) will be undertaken. The quantitative phases of the study will follow a prospective, cross-sectional design [17], while the qualitative phases will be phenomenological in nature, aiming to explore the experiences of participants [18]. The methodology for each of the 3 phases will be presented separately.

#### Phase 1: Developing and Obtaining Accreditation for a Digital Health Care Curriculum

During phase 1 of the study, the researchers aim to address 3 subobjectives to meet the main objective of this phase. The 3 subobjectives will be discussed separately, as their design and data collection procedures differ (Textbox 1).

**Curriculum development.**

**Subobjective 1**

Determining digital health care content and gaps in the current curriculum using gap identification frameworks when reviewing and analyzing curricula within a Health Care Sciences School.

- **Study design:** A quantitative, retrospective document review will be undertaken.
- **Sample:** A total of 6 published curricula from the Departments of Physiotherapy, Occupational Therapy, Human Nutrition and Dietetics, Nursing Sciences, and Speech-Language Pathology and Audiology will be included for review. Curricula from other departments or those that have not been published will be excluded from the review.
- **Data collection:** Each curriculum will be reviewed by the research team using the READ approach. The competencies, skills, and attitudes questionnaire [19] will be used to capture the data. Descriptive statistics will be applied to analyze the data.

**Subobjective 2**

Investigating the knowledge, skills, attitudes, and graduate attributes to be included in a digital health curriculum for students in a Health Care Sciences School.

- **Study design:** A prospective Delphi technique [19] will be undertaken.
- **Sample:** Potential participants for the Delphi expert panel will be identified through author and reference lists that will be compiled from a literature review, as well as referrals from academic directors and members of the academic community. To ensure diversity and representativeness in the participant group, a purposive sampling method will be used. Expert panel members will be considered for inclusion if they have published at least 2 or more papers or book chapters on digital health care, and if they are health care professionals. Experts with competing interests (such as digital health care company owners) will be excluded from participation.
- **Data collection:** Experts in digital health care (both national and international) who meet the eligibility criteria will be invited to participate in the study. Email invitations will be sent to all identified experts to participate in the Delphi study. Once consent is obtained, the first round of the survey will be sent via a Google link. Participants will have the option to add additional topics not included in the first-round survey, which will be added in the second round. After the first round, participants will be provided with the average ratings for each topic from the previous round. The second and third rounds will involve rating, scoring, and reaching a consensus on the survey items. An 80% agreement will be considered as consensus.

**Subobjective 3**

Developing a digital health care curriculum for a Health Care Sciences School in terms of module outcomes, content, teaching strategies, assessment outcomes, and submitting for evaluation and accreditation.

- **Study design:** An action-based approach to curriculum development will be undertaken [20].
- **Sample:** Task-team members, consisting of the researchers participating in the study will be included.
- **Data collection:** Information collected from the document review and the Delphi technique will be combined to establish the syllabus. A curriculum matrix will be developed based on the syllabus and will be populated with learning outcomes, assessment criteria, and assessment methods. The curriculum will be developed in accordance with the guidelines of the Health Professions Council of South Africa and the Council of Higher Education (South Africa). Once the curriculum is approved, it will be implemented. After six months of implementation, it will be assessed and changes will be made as needed.

#### Phase 2: Developing a Teaching, Learning, Clinical, and Research Center Situated Within a Health Care Sciences School

The second phase of the study aims to address 2 subobjectives in order to achieve the main objective for the phase (Textbox 2).

**Establishing a digital health care center.**

**Subobjective 1**

Conducting a readiness and needs analysis among staff and health care professionals regarding digital health care training needs.

- **Study design:** A quantitative, prospective survey design will be undertaken.
- **Sample:** All staff members associated with a Health Care Sciences School at a selected university will be included. Staff members will be selected for participation if they are part-time or full-time academic staff in the following departments: Physiotherapy, Occupational Therapy, Human Nutrition and Dietetics, Nursing Sciences, and Speech-Language Pathology and Audiology. To ensure a 50% distribution with a 5% margin of error and a 95% CI, 184 participants are required. All staff members in the School of Health Care Sciences will be targeted for inclusion to account for noncompletion of surveys and attrition during the study.
- **Data collection:** After obtaining permission from the Dean, an email with information and consent forms will be sent to all relevant staff members in the school. The questionnaire will be adapted from Bingham et al [21]. The link to the questionnaire will be emailed to all academic staff members. Once a questionnaire has been completed, access to the questionnaire will be terminated to prevent multiple submissions from the same participant. Descriptive analysis will be conducted on the collected data.

**Subobjective 2**

Developing a care services plan for the digital health care center and reviewing and consulting on existing technology and business plans to develop and validate the proof of concept for the establishment of a digital health care ecosystem.

- **Study design:** A qualitative explorative design will be undertaken.
- **Sample:** Snowball sampling will be conducted with at least 6 academic staff members from each department (refer to subobjective 1 for the list of departments), and data collection will cease once saturation is reached. Academic staff members with experience in clinical planning and involvement in health services programs will be included.
- **Data collection:** Staff members will be invited to participate in focus group interviews after obtaining permission from the Dean. A focus group interview schedule with open-ended questions will be used. The interviews will be voice-recorded and transcribed verbatim. Thematic analysis will be conducted using Tesch’s eight-step model [22].

#### Phase 3: Evaluation of the Impact of Community Engagement in the Design, Establishment, Content, and Delivery of Digital Health Promotion Projects Offered Through the Digital Health Care Ecosystem

The third and final phase of the study is divided into 2 subobjectives (Textbox 3).

**Community engagement.**

**Subobjective 1**

Evaluating the health needs of the local community.

- **Study design:** A cross-sectional, descriptive survey design will be undertaken.
- **Sample:** All relevant community stakeholders, including medical doctors, traditional healers, community leaders, and community health workers, will be considered for inclusion in the study. Purposive and network sampling will be used to identify as many relevant parties as possible for inclusion in the study. All community stakeholders, who have resided in the community surrounding the institution where the study will be conducted for at least 2 years, will be included. Community stakeholders with a conflict of interest (such as financial interests in the project) will be excluded from participation.
- **Data collection:** Known community stakeholders will be recruited for participation, and network sampling will be used to identify as many stakeholders as possible. Questionnaires will be circulated electronically to all participants. The questionnaires will be an adapted version of the Cooper University Health Care Health Assessment Survey [23]. Once a questionnaire is completed, access will be terminated to prevent multiple submissions. Descriptive analysis will be conducted on the collected data.

**Subobjective 2**

Exploring digital health care attitudes and service suggestions of shared roles and responsibilities of community members that can be implemented through digital health care in the specific community.

- **Study design:** A qualitative explorative design will be undertaken.
- **Sample:** All relevant community stakeholders (as given in subobjective 1) will be considered for participation. Purposive and network sampling will be used to identify as many relevant parties as possible for inclusion in the study. Data collection will cease once saturation is reached.
- **Data collection:** Community stakeholders will be invited to participate in focus group interviews, using a schedule with open-ended questions. The interviews will be recorded and transcribed verbatim. Thematic analysis will be conducted following Tesch’s eight-step model [22].

### Recruitment

The population from the institution and its surrounding areas will be recruited for the study. Staff and students from the health sciences institution will be recruited from the following departments: Departments of Physiotherapy, Occupational Therapy, Dietetics and Human Nutrition, Public Health, Nursing Sciences, and Speech and Language Pathology and Audiology. The student population will consist of undergraduate students who are enrolled at the Health Sciences institution. All part-time and full-time staff members employed at the institution, who do not have a financial interest in participating, will be included in the study. Community stakeholders recruited for the study will be restricted to a 30-kilometer radius from the institution. Recruitment will be conducted through email communication, flyer distribution within the institution and surrounding community, and word of mouth.

### Statistical Analysis

Due to the vast nature of the study, statistical analysis will vary for each subobjective. Quantitative data will be analyzed using SPSS (version 27; IBM Corp) software, while qualitative data will be analyzed using NVivo (Lumivero). To ensure optimal analysis of the qualitative data, Tesch’s eight-step method of data analysis will be conducted before uploading the data into NVivo. Data will be prepared by transcribing all interviews verbatim. Coding will be conducted on data segments that share similar themes. Thereafter, categories and coding schemes will be developed based on the identified themes from a sample of the text. The coding scheme will first be tested on a relatively small sample to ensure that the scheme is applicable before being applied to the entire dataset. A researcher, who will not be involved in the initial development of the coding scheme, will assess coding consistency, after which conclusions will be drawn from the coded data [22].

### Data Exclusion

For all quantitative phases of the study, data will be excluded from analysis if the dataset is incomplete (ie, if questionnaires are not fully completed). Data from all phases of the study will also be excluded if participants withdraw voluntary informed consent.

### Validity and Reliability

The questionnaires that will be used in this study are adapted from previously developed and tested questionnaires, with additional questions drawn from the literature [19,21,23]. The content validity of the questionnaires will be conducted to ensure that the questionnaires have a fair representation of the constructs to be measured. Construct validity will be ensured by providing evidence that the theoretical structure of the questionnaire is relevant. Face validity will ensure that the questionnaire is transparent and relevant, as it appears to elicit information from the participants. A pilot study will be conducted with the aim of ensuring that the data collection tools are reliable. Construct and face validity will be determined by asking 10 health care professionals from different institutions across the country to provide feedback to the researchers. Health care professionals will be identified through purposive sampling to ensure that they have the necessary expertise regarding digital health care. Reliability will be determined by assessing whether the questionnaires answer the stated research questions, whether the questions are clear and unambiguous, and whether the language and terminology used are easy to understand. Following the pilot study, the questionnaires will be revised if needed.

### Trustworthiness

Credibility will be ensured by building rapport and trust with the participants. The participants will be engaged in interviews to gain insights and understand their views. The researchers will ensure that honesty is maintained by giving information regarding the study to the participants. Dependability will be ensured by making the voice recorder and transcripts available to an external auditor for verification. Conformability and credibility will be ensured by conducting interviews until saturation is reached. Transferability is the ability to transfer research findings from one context to another [24]. The researchers will provide a thorough description of the research methods and setting in the research publications or any form of dissemination so that the applicability of the data to another context can be evaluated.

### Ethical Considerations

As human participants will be used during the course of the study, ethical approval for the conduction of the study was obtained from the Sefako Makgatho Health Sciences University Research and Ethics Committee (SMUREC/H/260/2023:PG). Participants in all phases of the study will be provided with information pertaining to the phase that they are participating in, before being asked to sign a voluntary informed consent. Participants hold the right to withdraw from the study without providing reasons, and without prejudice. All data collected will be anonymized to ensure participants’ privacy, and data will be stored in an encrypted electronic folder accessible only to the researchers. During the dissemination of results, the researchers will ensure that no identifying information is published. Participants in the study, regardless of the phase, will not receive remuneration for their participation. The research will be conducted in line with the regulations set forth by the Declaration of Helsinki.

## Results

Phase 1 of the study will be conducted from January to December 2024. The second and third phases will be conducted concurrently, from January 2025 to December 2026. A detailed schedule of the study is provided in Table 1 below.

**Table 1:** Projected time frames of the study.

Phase	Subobjective	Timeline
1	To determine digital health care content and gaps in the current curriculum by applying the gap identification framework when reviewing and analyzing curricula within a Health Care Sciences School.	January-June 2024 (completed)
1	To investigate which knowledge, skills, attitudes, and graduate attributes should be included in a digital health curriculum for students in a Health Care Sciences School.	April-October 2024 (completed)
1	To develop a digital health care curriculum for a Health Care Sciences School, in terms of module outcomes, content, teaching strategies and assessment outcomes, and submit it for evaluation and accreditation.	October-December 2024 (completed
2	To conduct a readiness and needs analysis among staff and health care professionals regarding digital health care training needs.	January-April 2025
2	To develop a care services plan for the digital health care center and to review and consult on existing technology and business plans to develop and validate proof of concept for the establishment of a digital health care center within a Health Sciences School.	May-December 2025
3	To evaluate the health needs of the local community.	January-June 2025
3	To explore digital health care attitudes and service suggestions, as well as shared roles and responsibilities of community members that can be implemented through digital health care in the specific community.	July-October 2026
4	Final write-up of all information collected in the study	October-December 2026

## Discussion

### Principal Findings

The establishment of digital health care ecosystems in tertiary institutions has the potential to have a significant positive influence on health care delivery in a country [2-6]. To successfully establish a digital health care ecosystem, it is essential to develop relevant curricula. Curricula represent an institution’s vision and mission and are expected to be contextually relevant and globally responsive to the diverse needs of communities [25]. A digital health care curriculum will ensure the long-term viability of such ecosystems, and studies have shown that both academics and students agree that the integration of digital health ecosystems is long overdue [26,27]. The proposed changes to the curricula will inevitably alter clinical training opportunities for students to ensure the development of digital health care competence.

The introduction of clinical digital health care training into formal education allows students to participate in areas such as interprofessional education, offering care to rural and remote areas (including exposure to international contexts), as well as improving their digital literacy and awareness of trends within the Fourth Industrial Revolution [8,28,29]. Literature indicates that the theoretical and practical components are connected and their combination ensures that students acquire not only the knowledge but also the clinical skills, competencies, attitudes, and important graduate attributes to provide excellent patient care [30,31].

In the development of digital health care curricula and clinical training, partnerships with community stakeholders are important to ensure that the integration of technology will empower community members [32]. Communities must be involved in the conceptualization, technology innovation, development, implementation, and monitoring of the health care system to ensure health care democracy and sustainability [32,33]. Well-structured community engagement has the potential to minimize the gap between research and policy by ensuring support from key stakeholders [34]. In addition, community involvement is an essential aspect of good health governance [35].

Due to the geographical limitations of the study (ie, recruitment of participants from a 30 km radius of the institution where the study will be conducted), the results that are obtained from the study may not translate to other health care institutions. However, the principles followed in the design and establishment of the digital health care ecosystem may be universally applicable.

### Conclusions

The establishment of a comprehensive digital health care ecosystem will be used for providing digital health care knowledge, conducting clinical training with students using a variety of digital health care approaches and technology, and conducting research into digital health care. The center aims to encourage stakeholder collaboration, provide educational opportunities, stimulate research, and provide service delivery using digital health care services. Important activities, such as interprofessional collaboration and community engagement, will be facilitated through the establishment of a digital health care ecosystem. The center would offer multidisciplinary and transdisciplinary health care services to multiple rural and remote contexts, including schools, old age homes, hospitals, and homes.

## Abbreviations

HCP: health care professional
WHO: World Health Organization

## Data Availability

Datasets generated or analyzed during the study are available from the corresponding author on reasonable request.
